# Fibril formation and therapeutic targeting of amyloid-like structures in a yeast model of adenine accumulation

**DOI:** 10.1038/s41467-018-07966-5

**Published:** 2019-01-08

**Authors:** Dana Laor, Dorin Sade, Shira Shaham-Niv, Dor Zaguri, Myra Gartner, Vasantha Basavalingappa, Avi Raveh, Edward Pichinuk, Hamutal Engel, Keita Iwasaki, Tatsuyuki Yamamoto, Hemanth Noothalapati, Ehud Gazit

**Affiliations:** 1 Department of Molecular Microbiology and Biotechnology, George S. Wise Faculty of Life Sciences, Tel Aviv University, 69978 Tel Aviv, Israel; 2 BLAVATNIK CENTER for Drug Discovery, Tel Aviv University, 6997801 Tel Aviv, Israel; 3 Faculty of Life and Environmental Science, Shimane University, Matsue, 690-8504 Japan; 4 Raman Center for Medical and Biological Applications, Shimane University, Matsue, 690-8504 Japan; 5 Department of Materials Science and Engineering, Iby and Aladar Fleischman Faculty of Engineering, Tel Aviv University, 69978 Tel Aviv, Israel

## Abstract

The extension of the amyloid hypothesis to include non-protein metabolite assemblies invokes a paradigm for the pathology of inborn error of metabolism disorders. However, a direct demonstration of the assembly of metabolite amyloid-like structures has so far been provided only in vitro. Here, we established an in vivo model of adenine self-assembly in yeast, in which toxicity is associated with intracellular accumulation of the metabolite. Using a strain blocked in the enzymatic pathway downstream to adenine, we observed a non-linear dose-dependent growth inhibition. Both the staining with an indicative amyloid dye and anti-adenine assemblies antibodies demonstrated the accumulation of adenine amyloid-like structures, which were eliminated by lowering the supplied adenine levels. Treatment with a polyphenol inhibitor reduced the occurrence of amyloid-like structures while not affecting the dramatic increase in intracellular adenine concentration, resulting in inhibition of cytotoxicity, further supporting the notion that toxicity is triggered by adenine assemblies.

## Abstract (web summary)

Small molecule metabolites like phenylalanine can form amyloid-like structures but so far this has only been demonstrated in vitro. Here the authors generate a yeast in vivo model of adenine self-assembly and characterize the adenine assemblies in cells by indicative amyloid dye and anti-adenine assemblies antibodies.

## Introduction

The canonical amyloid hypothesis attributed the formation of nano-scale fibrillar assemblies exclusively to proteins and polypeptides^1,2^. However, a paradigm for the pathophysiology of inborn error of metabolism disorders significantly extended the original hypothesis, showing that at millimolar pathological concentrations, the single phenylalanine amino acid can form nanofibrillar structures in aqueous solution and neutral pH in vitro^3^. These nonproteinaceous assemblies exhibit typical apple-green birefringence and clear fluorescence signal upon Congo red staining when examined under cross-polarized light and fluorescent microscopy, intense fluorescence following thioflavin T staining, and cell culture cytotoxicity^3,4^. Using electron microscopy, a fibrillar morphology of the phenylalanine assemblies was observed, showing physical properties characteristic of protein amyloids. As opposed to single crystals that show regular geometrical shapes consisting of flat faces, amyloid structures have a fibrillar morphology. Based on the similar characteristics to amyloid proteins, these nonproteinaceous assemblies were suggested to display ‘amyloid-like’ properties.

The notable toxicity of the assemblies was suggested to be associated with the neurological damage observed in non-treated patients suffering from the phenylketonuria (PKU) error of metabolism disorder, in which phenylalanine accumulates due to metabolic pathway alteration. Histological post-mortem staining of brain tissues of human PKU patients, as well as of PKU model mice, using specific antibodies raised against phenylalanine fibrils, demonstrated the specificity of the antibodies and the formation of metabolite amyloid-like assemblies in the disease state^3^. Follow-up studies supported the notion that the single phenylalanine amino acid can form amyloid-like nanofibrillar structures, established the mechanism of oligomerization, and determined the ability of the phenylalanine assemblies to interact with phospholipid membranes, similar to protein amyloids^5–13^. Furthermore, doxycycline, epigallocatechin gallate, and tannic acid (TA), known inhibitors of amyloid fibril formation, were shown to counteract both phenylalanine aggregation and cytotoxicity of the assemblies in vitro^14,15^. Moreover, the amyloid hypothesis was significantly extended by demonstrating that several other metabolites, including additional amino acids and nucleobases, could form such archetypical nanofibrils in vitro, displaying amyloid-like properties^4,16–21^. The alanine amino acid shows none of the above characterizations, as well as no toxic effect when added to cultured cells at high concentrations^3,4^. Furthermore, differential flexibility properties might explain the resistance of alpha-phenylglycine, that differs from phenylalanine by the absence of an additional flexible carbon extension, to fibril formation^12^. Thus, fibril formation and toxic effect are believed to occur due to structures formed by only certain metabolites.

Inborn errors of metabolism, stemming from mutations resulting in enzymatic deficiencies in various metabolic pathways, can lead to the accumulation of substrates. Thus, for example, the required daily allowance (RDA) of phenylalanine for the general population may actually be toxic to individuals with PKU. Therefore, in the absence of strict dietary restrictions, PKU can lead to mental retardation and other developmental abnormalities. The recent extension of the amyloid hypothesis offers opportunities for both diagnostics, as well as therapy of these disorders. Specifically, inborn mutations in genes involved in the adenine salvage pathway in humans can lead to the development of several metabolic disorders as a result of the accumulation of adenine and its derivatives^22,23^. We have previously shown the formation of adenine amyloid-like structures in vitro. These assemblies displayed amyloidogenic properties, including the appearance of typical amyloid fibrils as demonstrated by electron microscopy, positive staining with amyloid-specific dyes, and notable cytotoxicity in cultured cells^4^. Moreover, formation of the adenine structures was shown to be inhibited by amyloid-specific inhibitors in vitro and adenine assemblies could interact with a membrane model, similar to their proteinaceous counterparts^15,24^. Yet, analysis of the formation of amyloid-like assemblies by metabolites has so far been limited to in vitro studies. Thus, there is a genuine need for in vivo models for the formation of such assemblies in order to understand the biological relevance and the consequences of metabolite molecular self-assembly. Yeast can assist in revealing the core abnormal processes underlying multiple aspects of biomolecular aggregation^25^. The pioneering work of Susan Lindquist and coworkers, as well as follow-up studies, had established yeast as an excellent model for several amyloid-associated disorders, including Alzheimer’s disease^26^, Parkinson’s disease^27,28^, Huntington’s disease^29,30^, and prion disorders^31^ and recently also type 2 diabetes^32^, supporting the high relevance of this approach for further establishing the extended amyloid hypothesis. Expression of mutated genes involved in amyloid diseases in yeast was previously shown to result in a decrease in yeast growth rate, suggesting a toxic effect, similar to that observed in neurons. Furthermore, yeast offer an attractive platform by virtue of their genetic tractability, known homeostasis mechanisms, ease of manipulation and short generation time, facilitating screens and the development of agents for disease-modifying therapy^25,28^. Here, we established an in vivo model for the study of the self-assembly of adenine into amyloid-like structures. Our study provides a methodology for establishing additional yeast models that could most reliably mimic the metabolic state in inborn error of metabolism disorders, as the native metabolic pathways may be manipulated with no artificial introduction of genes.

## Results

### In vivo model for adenine accumulation and toxicity

**Fig. 1:**
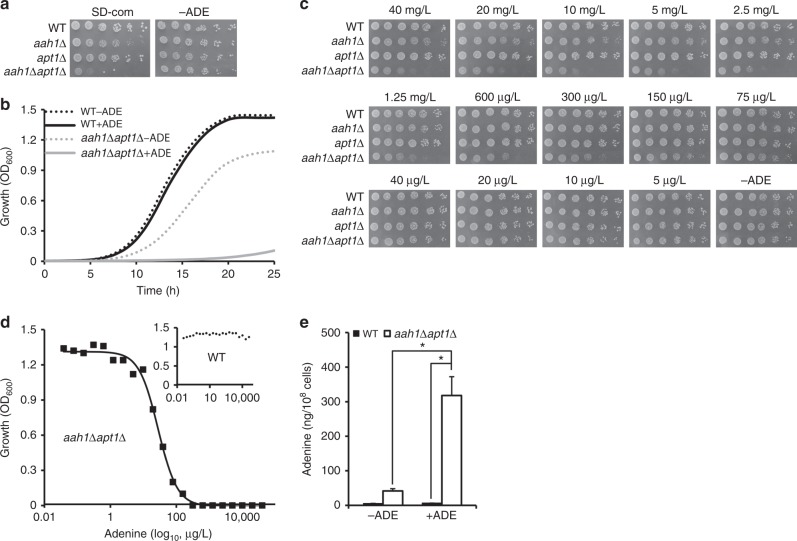
Sensitivity of the salvage mutant to adenine feeding. **a** Wild-type (WT), *aah1*Δ, *apt1*Δ, and *aah1*Δ*apt1*Δ strains were serially diluted and spotted on SD complete medium containing 20 mg/L adenine (SD-com) or on SD medium without adenine (−ADE). **b** Growth curves of WT and *aah1*Δ*apt1*Δ cells in SD media with or without adenine (ADE). SD-com is denoted as +ADE to emphasize the presence of adenine in the standard yeast growth medium. **c** WT, *aah1*Δ, *apt1*Δ, and *aah1*Δ*apt1*Δ strains were serially diluted and spotted on SD medium with various concentrations of adenine, ranging from 40 mg/L (~300 µM) to 5 µg/L (~0.0375 µM), or without adenine (−ADE). **d** Cells were grown in the presence of different concentrations of adenine, ranging from 40,000 µg/L (~300 µM) to 0.038 µg/L (~0.029 nM), in SD medium and the absorbance at 600 nm was measured at the logarithmic phase. The results were fitted using a four-parameter logistic equation (4PL), *R*^2^ > 0.99. Inset shows the absorbance of the WT at 600 nm. **e** Intracellular concentration of adenine determined using GC–MS. WT and *aah1*Δ*apt1*Δ cells were grown in SD media in the absence (−ADE) or presence (+ADE) of adenine and the metabolites were extracted. *P < 0.01 (Student’s *t*-test). Values are the mean ± s.d. of three experiments

Purine biosynthesis pathways are crucial for the normal function of cells and are conserved between yeast and humans. Both the adenine phosphoribosyltransferase (APRT) and adenosine deaminase (ADA) enzymes take part in adenine salvage in humans and mutations in their encoding genes can lead to the accumulation of adenine and its derivatives^22,23^. Mutation in APRT leads to the APRT deficiency and mutation in ADA leads to ADA deficiency. In budding yeast, the APRT and ADA orthologs (*APT1* and *AAH1*, respectively) are similarly involved in adenine salvage^33^. Apt1 catalyzes the formation of AMP from adenine and Aah1 converts adenine to hypoxanthine. To reveal the importance of these two enzymes for cell growth, we generated an adenine salvage mutant by disruption of both *APT1* and *AAH1* genes. The double mutant showed a slow growth phenotype on synthetic defined (SD) medium containing a specific mixture of amino acids and nucleobases (SD complete), compared to the growth of the wild-type and both single mutants (Fig. 1a, b). Interestingly, removal of adenine from the medium dramatically improved cell growth of the metabolite salvage mutant and had no significant effect on any of the other strains (Fig. 1a, b). The double mutant showed a slow growth phenotype also when using a minimal medium containing glycerol as a nonfermentable carbon source (Supplementary Fig. 1). Thus, the reduced growth of the salvage mutant depended only on the presence of adenine, regardless of the carbon source, ruling out the possibility that the salvage mutant had lost respiratory competence due to mitochondrial mutations. To verify that the toxic effect observed for the salvage mutant is indeed due to disruption of the *AAH1* and *APT1* genes, single copy plasmids carrying both genes were introduced to the mutant cells by transformation. As shown in Supplementary Fig. 2, the restoration of the genes indeed rescued the growth phenotype.

Altogether, the presence of adenine at the normal concentration used for wild-type yeast growth leads to a significant cell growth decrease in a strain that is defective in the biosynthesis downstream to adenine. This is indeed an unusual phenomenon as in most cases (excluding mutations in transport systems) the absence of a given metabolite, rather than its presence in the physiological concentration needed for normal yeast growth, serves as a limiting factor of wild-type yeast growth^34^. This is analogous to the toxicity of normal metabolite concentrations observed in some inborn error of metabolism disorders, such as PKU and tyrosinemia^35^, where the RDA for the general population is actually toxic to the affected individuals, due to accumulation of the metabolites in the absence of salvage. The effect indeed appears to reflect toxicity, as increasing adenine levels in the medium led to decreased cell growth in a dose-dependent manner (Fig. 1c, d), similar to the toxic effect previously observed in in vitro cell culture studies^4^. It should be noted that the toxic effect on cell growth due to adenine accumulation is very similar to the outcome of protein amyloid expression in yeast cells^26,28,29^.

### Non-linear response to adenine feeding

The dose–response curve of cell growth as a function of adenine concentration was fitted using a four-parameter logistic equation (4PL), producing a typical-sigmoidal shaped curve, with no effect at lower concentrations and a sharp increase in the inhibitory effect upon reaching a critical concentration threshold (Fig. 1d). Thus, cell growth appears to be affected by adenine levels in a non-linear cooperative manner. This is consistent with the mechanism of nucleation-growth as observed in micelle formation (assembly above a critical micelle concentration, CMC) or the formation of amyloids by the assembly of protein monomers^36–38^. Indeed, in vitro studies also showed non-linear self-assembly of adenine at different concentrations (Supplementary Fig. 3 and Supplementary Methods). Thus, we present in vitro as well as in vivo data regarding adenine accumulation at different concentrations and the possible correlation between the assembly mechanism of fibril formation and cellular growth. Our results imply that above a critical concentration, the favorable energetic state dictates the formation of the toxic assemblies, while at lower concentrations the metabolites are most likely at the normal physiological state.

To quantify the intracellular concentrations of adenine, gas chromatography mass-spectrometry (GC–MS) was used. Analysis of the cellular adenine concentrations under different conditions indicated that on SD media in the presence of the metabolite, adenine levels were indeed significantly higher in the salvage mutant as compared to the wild-type strain (×45 fold; Fig. 1e), suggesting a clear correlation between the feeding amounts of adenine and growth inhibition. Similarly, when not following a very strict diet, inborn error of metabolism patients have 1–2 orders of magnitude higher concentrations of metabolites, as compared to the general population^10^.

### Visualization of adenine accumulation by Raman imaging

**Fig. 2:**
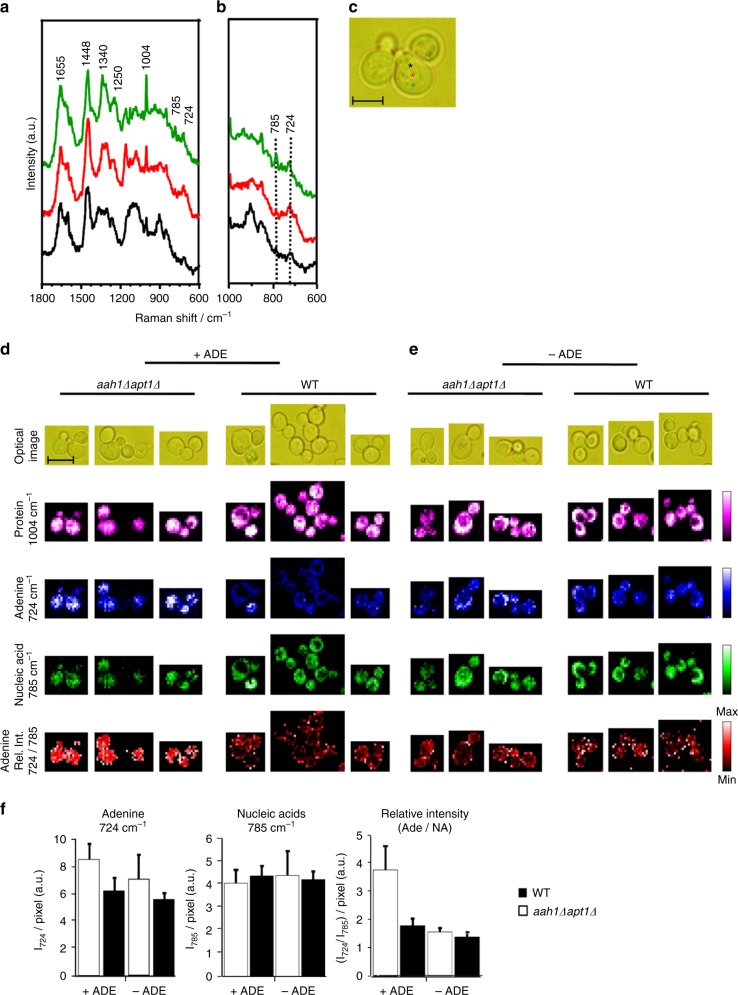
In vivo Raman visualization of adenine accumulation. **a**–**c** Space-resolved Raman spectra of mutant yeast (*aah1*Δ*apt1*Δ). Whole fingerprint region of the Raman spectrum (**a**) and zoom in of nucleic acids and the adenine marker region (800–600 cm^−1^) (**b**) are shown. The corresponding optical image is presented and the measured points are indicated using colored asterisks. Scale bar is 5 µm (**c**). **d** and **e** Raman chemical images of the salvage mutant and wild-type yeast cells in the presence of adenine (**d**) and in the absence of adenine (**e**). Raman images of protein (1004 cm^−1^; magenta), adenine (724 cm^−1^; blue), nucleic acids (785 cm^−1^; green) and the relative intensity of adenine and nucleic acids (724/785 cm^−1^; red) are presented. Corresponding optical images are included for reference. Rel. Int., relative intensity. Scale bar is 5 µm. **f** Calculated average intracellular intensity from yeast cells of 724 cm^−1^ per pixel, 785 cm^−1^ per pixel, and relative intensity of WT and *aah1*Δ*apt1*Δ yeast cells. *P* < 0.02 (Student’s *t*-test). Values are the mean ± s.d. of three experiments

To detect the accumulation of adenine in vivo, we utilized Raman microspectroscopy. Raman spectroscopy coupled with microscopy has recently emerged as a promising tool to trace intracellular processes in vivo and was successfully used in yeast to follow glucose assimilation into intracellular components^39^. Raman spectrum provides rich and highly specific chemical information and thus intracellular molecular distribution can be visualized at a sub-μm spatial resolution by Raman microspectroscopy. Moreover, being a vibrational spectroscopic technique, label-free imaging could be performed, as it requires no exogenous dye probe. Space-resolved Raman spectra obtained at three different points in salvage mutant single cells (*aah1*Δ *apt1*Δ) in the presence of adenine showed Raman bands characteristic to proteins (Fig. 2a, c). Major features included 1004 cm^−1^ [phenylalanine ring breathing], 1250 cm^−1^ [amide III], 1340 and 1448 cm^−1^ [C–H bend], and 1655 cm^−1^ [amide I]^40–42^. By carefully examining the region between 800 and 700 cm^−1^, we could observe a band at 785 cm^−1^ corresponding to ring breathing modes of nucleobases such as uracil, cytosine, thymine, and nucleic acid backbone vibration (Fig. 2b). Additionally, we also observed a band at 724 cm^−1^ corresponding to adenine ring breathing modes (Raman band of adenine in solid and solution forms are shown in Supplementary Fig. 4). According to these observations, we chose the 785 cm^−1^ band as a marker of nucleic acids in general while 724 cm^−1^ served as an adenine marker^43–45^. As expected, the intensities of these two bands varied depending on the location in the cell. As shown in Fig. 2b, while the red and black spectra showed intense adenine marker with very low nucleic acid band (indicating high prevalence of adenine), respectively, the spectrum in green showed comparable intensities of these two bands, which is typical of nucleic acids. Thus, these bands can be used to study adenine accumulation.

In order to further investigate adenine accumulation in living yeast cells, we performed Raman imaging experiments on both mutant and wild-type yeast in the presence (Fig. 2d) or absence (Fig. 2e) of adenine. We first imaged macromolecules such as proteins, using phenylalanine ring breathing mode at 1004 cm^−1^ (magenta). No significant difference was observed, indicating that proteins were similarly distributed under all conditions. In the presence of externally added adenine, images constructed using 724 cm^−1^ showed high intracellular abundance of adenine in the double mutants, while its distribution was very low in wild-type yeast (blue). In the absence of externally added adenine, the intensity of intracellular adenine was comparable in both mutant and wild-type cells. To exclude the contribution of adenine from nucleic acids, we calculated the relative intensity of 724 cm^−1^ images (blue) and nucleic acids 785 cm^−1^ images (green) (724/785 cm^−1^), as shown in red. Relative intensity images showed adenine accumulation and the appearance of subcellular regions of high adenine concentration only in double mutant yeast cells in the presence of adenine. Average intracellular intensity of 724 cm^−1^ per pixel, 785 cm^−1^ per pixel, and the relative intensity per pixel of WT and *aah1*Δ*apt1*Δ cells were calculated (Fig. 2f). The results reinforce the imaging results, showing significant adenine levels only in the mutant cells.

### Amyloid-like assemblies formation upon adenine accumulation

**Fig. 3:**
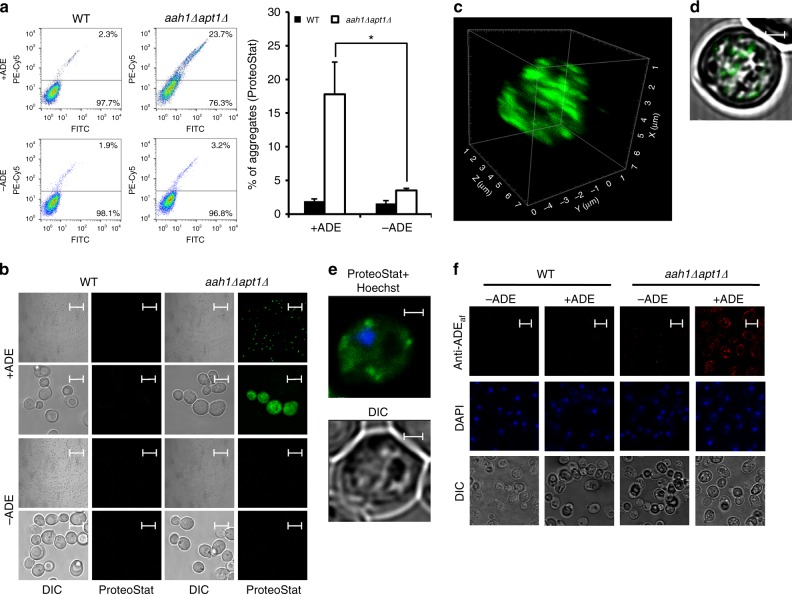
In vivo formation of amyloid-like structures upon adenine feeding. **a** and **b** Flow cytometry analysis (**a**) and representative confocal and differential interference contrast (DIC) images (**b**) of WT and *aah1*Δ*apt1*Δ cells under the indicated conditions using ProteoStat staining. *P < 0.01 (Student’s *t*-test). Values are the mean ± s.d. of three experiments. Scale bars are 50 and 5 μm for the lower (first and third lines) and higher resolution (second and fourth lines) images. **c** and **d** Z-stack followed by 3D reconstruction (**c**) and projection of a single section (**d**) of *aah1*Δ*apt1*Δ cells using ProteoStat. The analysis was performed using the Imaris software. Scale bar in (**d**) is 1 µm. **e** Representative image of *aah1*Δ*apt1*Δ cells double-stained with Hoechst and ProteoStat. Cells were visualized using DIC microscopy. Scale bar is 1 µm. **f** Representative confocal and differential interference contrast (DIC) images of WT and *aah1*Δ*apt1*Δ cells under the indicated conditions. Cells were fixed and subjected to indirect immunofluorescence using a polyclonal antibody raised against adenine amyloid-like assemblies, designated as Anti-ADE_af (=amyloid fibrils)_. Scale bar is 5 µm

To examine whether the observed non-linear behavior of the dose-dependent toxicity is indeed associated with self-organization of the metabolites into amyloid-like assemblies in vivo, the cells were stained with ProteoStat, an amyloid-specific fluorescent dye. This reagent was previously shown to facilitate specific and sensitive detection of amyloid aggregates in living cells^46^. To validate the possible identification of amyloid fibrils by ProteoStat staining in yeast, detection of the prion protein Sup35 was examined (Supplementary Fig. 5), showing significant and gradual increase in the staining of [*psi*^−^] compared to two types of [*PSI*^+^] aggregates, weak [*PSI*^+^] and strong [*PSI*^+^]. Flow cytometry and confocal microscopy were then employed to detect the presence of intracellular amyloid-like adenine aggregates. Both techniques clearly indicated the presence of amyloid-like structures in the adenine salvage mutant. Flow cytometry showed a higher degree of aggregation in the mutant compared to wild-type cells in the presence of the metabolite (Fig. 3a). Moreover, consistent with the indicated adenine sensitivity (Fig. 1a), removal of adenine from the medium reduced the level of aggregation in the mutant cells (Fig. 3a). Confocal microscopy further allowed the identification of the intracellular localization of the adenine aggregates, showing clearly stained dots only in the salvage mutant and specifically following the addition of adenine (Fig. 3b). Z-stack followed by 3D reconstruction (Fig. 3c), as well as projection of a single section of the Z-stack (Fig. 3d), showed that the stained dots were localized inside the cell, excluding the possibility of their attachment to the outer membrane or cell wall. Furthermore, Hoechst staining showed no co-localization, ruling out the possibility that the stained dots were inside the nucleus (Fig. 3e).

The Hsp104 chaperon was previously shown in yeast to play a pivotal role in the formation of numerous aggregates by structurally unrelated proteins and its deletion partially restored the viability of cells expressing Aβ and polyQ in yeast models for Alzheimer’s disease and Huntington’s disease, respectively^47,48^. To test whether the toxicity following adenine accumulation in the salvage mutant is mediated by Hsp104, cell growth upon adenine addition was examined in the presence of guanidine hydrochloride that was repeatedly shown to inhibit Hsp104 activity, as well as in a *hsp104*Δ mutant background (Supplementary Fig. 6a, b). No Hsp104 dependency was observed, suggesting that the toxicity induced by adenine accumulation involves a different mechanism than the Hsp104-associated one. To further validate this observation, the chaperon response was examined using a Hsp104-mCherry strain^49^ (Supplementary Fig. 6c). While Hsp104 recruitment and accumulation was observed under heat shock stress and in the presence of adenine both in wild-type and in the salvage mutant cells, no such dots appeared under optimal growth temperature, suggesting a different mechanism other than the Hsp104-associated one that was previously reported.

### In vivo formation of adenine amyloid-like assemblies

To confirm that the staining with the ProteoStat dye specifically identifies adenine amyloid-like structures, antibodies against adenine fibril structures were generated, as we have previously described for phenylalanine and tyrosine fibrils^3,50^. After staining with the anti-adenine fibril antibodies, fluorescent dots could be detected only in the salvage mutant in the presence of adenine (Fig. 3f). This result suggests that the observed cellular toxicity is directly linked to self-assembled amyloid-like adenine structures.

### Inhibition of adenine toxicity by TA

**Fig. 4:**
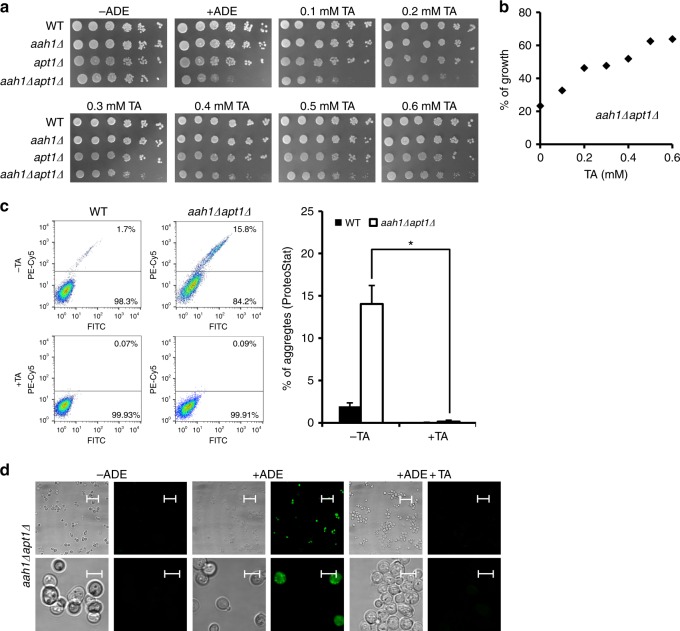
Addition of TA rescues the toxic effect observed in the adenine salvage mutant. **a** WT, *aah1*Δ, *apt1*Δ, and *aah1*Δ*apt1*Δ strains were serially diluted and spotted on SD medium without adenine (−ADE) or on SD media containing 2 mg/L adenine with or without various concentrations of TA, as indicated. **b** Dose–response curve for *aah1*Δ*apt1*Δ cells in SD medium containing adenine and TA at different concentrations. The percentage of growth represents the growth with TA compared to the growth without TA. **c** Flow cytometry analysis of WT and *aah1*Δ*apt1*Δ cells under the indicated conditions following ProteoStat staining. *P < 0.01 (Student’s *t*-test). Values are the mean ± s.d. of three experiments. TA concentration was 0.5 mM. **d** Representative confocal microcopy images of *aah1*Δ*apt1*Δ cells under the same conditions as in (**c**). Cells were visualized using DIC microscopy. Scale bars are 50 and 5 μm for the lower (first line) and higher resolution (second line) images

We next aimed to manipulate the formation of the intracellular adenine amyloid-like structures formed at high cellular concentrations of adenine upon feeding. Polyphenols comprise a large group of natural and synthetic small molecules, which were repeatedly shown to inhibit the formation of protein amyloid fibrils^51,52^, including the formation of aggregates that is associated with neurodegenerative diseases^53^. We examined the effect of TA, a widely studied polyphenol which was suggested as a potent inhibitor of β-amyloid fibrillation and of the assembly of the PrPsc prion protein^54,55^. Moreover, we have recently demonstrated the inhibition of adenine self-assembly in vitro by this polyphenol compound^15^. We found that in the presence of adenine, the addition of TA to the yeast media clearly improved cell growth of the salvage mutant in a dose-dependent manner (Fig. 4a, b). Staining of the cells with the ProteoStat amyloid-specific dye followed by flow cytometry allowed the detection of a decrease in metabolite aggregation in the presence of the inhibitor, despite the external addition of adenine to the media (Fig. 4c). Furthermore, the absence of the stained dots following the addition of TA was demonstrated by confocal microscopy (Fig. 4d). Based on mass-spectroscopy analysis (Supplementary Fig. 7a, b and Supplementary Methods), TA and adenine were detected in yeast cell debris as well as inside the cells, in yeast soluble material, indicating that TA enters the cells. To verify the molecular identity of the TA peak, we performed the same analysis on samples that contained adenine but not TA, showing only the peak corresponding to adenine (Supplementary Fig. 7c, d).

In order to ascertain that the inhibition by TA represents a general phenomenon applicable for other known amyloid inhibitors, baicalein, an additional polyphenolic inhibitor of protein amyloid formation, that was recently shown to bear therapeutic potential for Alzheimer’s and Parkinson’s disease^56^ was examined. Indeed, the use of this inhibitor resulted in a significant effect on yeast cell growth (Supplementary Fig. 8a, b) and on adenine aggregation (Supplementary Fig. 8c), as well as in a dose-dependent inhibition of adenine self-assembly in vitro (Supplementary Fig. 8d).

### TA mechanism of action

**Fig. 5:**
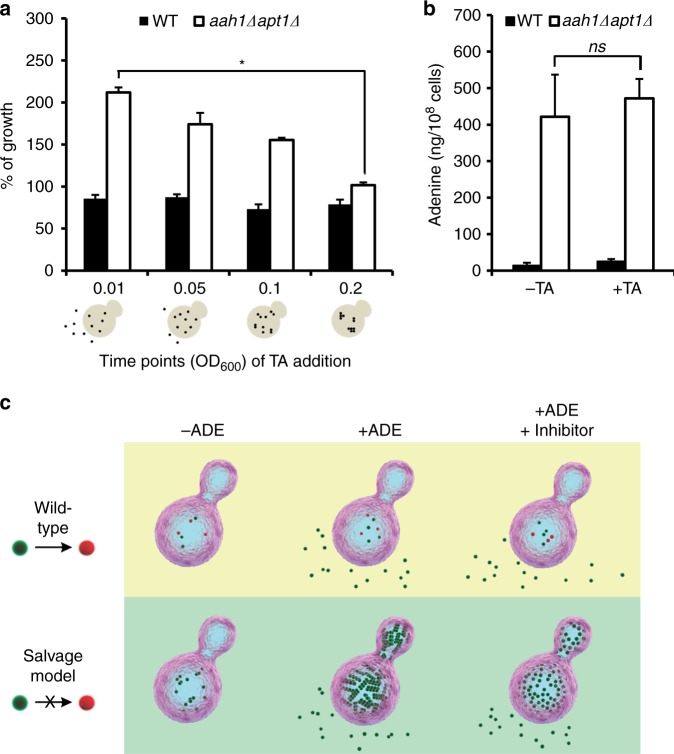
TA rescues salvage model yeast by preventing adenine assembly into toxic amyloid-like structures. **a** WT and *aah1*Δ*apt1*Δ cells diluted to OD_600_ 0.01 were grown in SD media in the presence of adenine. 0.5 mM TA was added to the samples at different OD_600_ values (0.01, 0.05, 0.1, and 0.2). *P < 0.01 (Student’s *t*-test). The percentage of growth represents the growth with TA compared to the growth without TA. Values are the mean ± s.d. of three experiments. Schematic illustrations of adenine assembly inside the cells at the different OD_600_ values following the addition of TA are shown below the *X*-axis. **b** Intracellular concentration of adenine determined using GC–MS. WT and *aah1*Δ*apt1*Δ cells were grown in SD media in the presence of adenine, with or without 0.5 mM TA, and the metabolites were extracted. ns, not significant (Student’s *t*-test). Values are the mean ± s.d. of three experiments. **c** Schematic model of adenine accumulation in WT cells compared to the adenine salvage model in the absence or presence of adenine, and following the addition of the TA inhibitor. The model reflects the relative amounts of the metabolite as determined experimentally. Upon feeding of the salvage mutant with adenine, the metabolite accumulates into ordered assemblies. Administration of the inhibitor, as recently shown by an in vitro study^15^, prevents the formation of assemblies at the nucleation phase, without any significant effect on the total concentration of the metabolite

In order to examine the in vivo mechanism underlying the inhibition of adenine amyloid-like formation by TA, the inhibitor was added at different time points of yeast growth. TA was found to hinder the formation of adenine amyloid-like assemblies when added at earlier time points of yeast growth, while it had no effect on their growth when added at later stages, suggesting that TA is most effective at the nucleation and early oligomerization stage of the metabolite self-assembly (Fig. 5a). These results further support a correlation between adenine aggregation into ordered structures and growth inhibition, suggesting that the toxic effect is induced by adenine accumulation into amyloid-like species. This effect can be rescued by either removal of adenine from the medium or addition of the amyloid inhibitor, thereby modulating the assembly process.

Finally, we examined whether the dramatic effect of the inhibitor on the viability of the mutant cells actually reflects a change in the concentration of adenine in the metabolite salvage model, in addition to the inhibition of amyloid-like structure formation. For this purpose, the intracellular concentrations of adenine with and without the inhibitor were compared using mass spectrometry. Evidently, while the addition of the inhibitor drastically improved cell growth, the intracellular levels of adenine remained constant (Fig. 5b). Thus, the change in cell growth appears to occur specifically due to the inhibition of adenine aggregate formation, and not as a result of a decrease in adenine levels, ruling out the possibility of an indirect effect of TA on adenine availability in the medium. This result, in addition to studies demonstrating the ability of TA to inhibit amyloid-like fibrillation of adenine in vitro^15^, further implies that the assemblies, rather than free metabolite molecules, mediate cell toxicity (as modeled in Fig. 5c).

## Discussion

The ability of metabolites to form ordered amyloid-like assemblies in vitro represents a significant extension to the “amyloid hypothesis” and provides a paradigm for the etiology of inborn error of metabolism disorders. The current study offers an in vivo demonstration and key experimental tools for the study of metabolite aggregation phenomena in living systems. One of the most intriguing results is the non-linear dose-dependency of the salvage mutant growth inhibition upon external addition of adenine (Fig. 1d). This type of cooperative behavior is typical of the well-coordinated self-assembly processes of protein and peptide amyloids. Significant effort was invested in order to understand the organization of amyloids formed by the assembly of various disease-related protein building blocks including the deciphering of primary and secondary nucleation events and the resulting fibril growth^57,58^. While these highly important studies were performed in vitro, the analysis of such processes in living cells has so far been hampered. The established model could provide an invaluable, yet simple, in vivo system to determine amyloid nucleation and assembly in living eukaryotic cells. In the current work (Fig. 5c), adenine aggregation and the formation of adenine amyloid-like structures inside the cell were demonstrated, as well as the suppression by well-known amyloid inhibitors.

The stereotypical cell-to-cell cerebral spreading of neurodegenerative disorders, such as Alzheimer’s disease, Parkinson’s disease, and amyotrophic lateral sclerosis (ALS) has recently been suggested to be propagated in a prion-like manner^59–61^. Given the remarkable similarities between protein and metabolite amyloids, prion-like features of metabolite assemblies cannot be ruled out. In fact, such a behavior may provide insights into surprising phenomena, including the maternal error of metabolism disorders, in which the newborn has normal metabolism but might still suffer from neurodevelopmental aberrations due to maternal effects^10,62^. Yeast have been successfully used to study cell-to-cell spreading of yeast prions^63,64^. The current system should allow to reveal any possible prion-like effects of nonproteinaceous aggregates. This will extend not only the definition of amyloids, but also the definition of metabolite prion-like entities, or ‘metions’ (*met*abolite *in*fectious particles).

The yeast model could also serve as the perfect platform for high throughput screening of therapeutic agents to target metabolite aggregation, as previously demonstrated for yeast models of protein amyloid self-assembly^28^. Yeast mutations, resulting in the accumulation of given metabolites, as observed in various human metabolic diseases, may be utilized in a simple yet robust way. The ability of TA to reverse the growth inhibition without an effect on metabolite concentration (Fig. 5b) provides a proof of concept for a large-range screen of potential drugs that could target the aggregation process, as well as possible nucleating seeds.

Finally, the current work provides a clear indication that the assembly of metabolites into ordered amyloid-like structures, rather than merely their amount, mediates growth inhibition. Indeed, excessive concentrations of adenine caused a complete growth inhibition, which was rescued with pharmacological intervention. This further raises the question regarding the natural mechanisms that help unicellular and multicellular organisms to avoid aggregation upon temporary or chronic surge in the concentration of a given metabolite. Presumably, similar to the proteostasis machinery^65,66^, there are cellular mechanisms aimed to avoid metabolite aggregation or clear preformed metabolite nuclei. Such a ‘metabostasis’ mechanism appears to be crucial, as many of the essential components of life, including aromatic amino acids and purine nucleobases, appear to be highly aggregative. The use of these molecular components is essential to maintain functional biological systems, while safety mechanisms should be available to prevent unwanted association of these aggregation-prone entities.

## Methods

### Strains and culture

Yeast strains, plasmids, and primers used in this study are listed in Supplementary Tables 1, 2 and 3, respectively. Strains were cultured in SD media consisting a defined mixture of amino acids and nucleobases. Adenine (adenine hemisulfate salt, Sigma-Aldrich) was supplemented in the indicated concentrations.

### Construction of *aah1*Δ*apt1*Δ strain

The *kanMX6* cassete in the *apt1::kanMX6* strain was replaced by a PCR product of the HygromycinR cassette using U2/D2 oligonucleotides that contain a homology sequence for homologous recombination. Cells were grown in rich medium (YPD) for ~24 h before plating on YPD plates containing Hygromycin (200 mg/L). The double mutant strain *apt1::hphMX6 aah1::kanMX6* was generated by transforming a PCR product of the KanMX cassette with 5′ and 3′ flanking sequences of *AAH1* into the *apt1::hphMX6* strain. Cells were grown in YPD for ~24 h before plating on YPD plates containing G418 (200 mg/L). Genetic disruptions were confirmed by PCR using an oligonucleotide upstream of the deletion and a reverse oligonucleotide within the HygR or the KanMX gene.

### Yeast growth assays

Strains were grown over night at 30 °C in SD medium without adenine. For spotting assays, strains were diluted to 6.25*10^7^ cells/mL and were then five-fold serially diluted and spotted on SD media with the indicated concentrations of adenine or TA (Sigma-Aldrich). Plates were incubated at 30 °C for 2 days. The results displayed are representative of three biological experiments.

For OD_600_ measurements, strains were diluted to OD_600_ 0.01. 200 µL of cells were platted on 96 wells plates and incubated at 30 °C for 25 h with continuous shaking. OD_600_ was measured using Tecan^TM^ SPARK 10M plate reader. The results displayed are representative of three biological experiments performed in triplicate.

For the dose–response curve of WT and *aah1*Δ*apt1*Δ cell growth (Fig. 1d), strains were cultured in SD media containing different concentrations of adenine (from 0.03 µg/L to 40 mg/L) and OD_600_ measurements were performed when the cells reached log phase (1*10^7^ cells/mL). The results displayed are representative of three biological experiments. Results were fitted to a standard logistic four-parameter equation using the OriginLab software using the following equation:

1 $${y} = {d} + \frac{{{a} - {d}}}{{\left( {1 + \left( {\frac{{x}}{{{c}^{b}}}} \right)} \right.}}$$

where *y* is the OD_600_ value, *d* is the maximum OD_600_ value, *a* is the minimum OD_600_ value, *x* is adenine concentration (µg), *c* is the point of inflection, and *b* is the slope factor of the curve.

For the percentage of growth analysis (Figs. 4b and 5a), OD_600_ was measured when cells reached log phase. The percentage of growth represents the growth with TA compared to the growth without TA. The results displayed are representative of three biological experiments performed in triplicate.

### GC–MS

The protocol was adapted from Tu, B. P. et al.^67^. Four milliliters of 60% methanol/10 mM Tricine (pH 7.4) that was maintained at −20 °C were added to 2 mL of logarithmic cells. The number of cells was counted using a hemocytometer. Cells were incubated for 5 min at −20 °C, centrifuged at 1000×*g* for 3 min at 4 °C, washed with 1 mL of the same buffer and resuspended in 1 mL of 75% ethanol/0.5 mM Tricine (pH 7.4). Intracellular metabolites were extracted by incubating at 80 °C for 3 min and then at 4 °C for 5 min. Samples were centrifuged at 20,000×*g* for 1 min, and 0.9 mL of the supernatant was transferred to a new tube, centrifuged again for 10 min and 0.8 mL was transferred to a new tube. Samples were stored at −80 °C until analysis. For each strain and/or condition, seven samples were extracted: three samples for measurement and four samples for spiking adenine standard. Matrix-matched standard curves for each cell pool were constructed^68^. Stock solution of adenine (1 mg/mL in 1 M HCl) was sequentially diluted 10-fold, 100-fold, and 1000-fold into solutions that were spiked to four samples of the same group of extracts plus one blank in amounts providing final exogenous adenine concentrations of 1, 5, 10, and 50 µg/mL after derivatization. All samples were evaporated in N_2_-flow and then lyophilized for 2 h. Each sample was derivatized by adding 30 µL of MSTFA (Restek, #35600) and shaking at 60 °C for 1 h. The mixtures were transferred to 2 mL autosampler glass vials with 100 µL glass inserts. Acquisition was performed in GC–MS system comprising an Agilent 7890A gas chromatograph and LECO Pegasus HT Time-of-Flight Mass Spectrometer (MassBank Record: OUF00096) using a Rxi-5Sil MS (Restek) column. Samples were analyzed using the splitless mode; the temperature of injector was set to 230 °C and the transfer line to 250 °C. The injected analytes were separated using the following chromatographic conditions: 1 mL/min of helium as a carrier gas was held at 80 °C for 2 min, ramped to 330 °C at 15 °C/min, and then held at this temperature for another 6 min. For mass-spectrometry, electron impact mode with ionization voltage was 70 eV, mass range 85–500*m*/*z* and acquisition rate of 20 spectra per second. The ion source chamber was set to 200 °C, and the detector voltage was 1650V. LECO ChromaTOF software was used for acquisition control and data processing with 264*m*/*z* at 722-s peak for quantitation, with the limit of quantitation being 0.01 µg/mL. Adenine concentration was normalized to the number of cells per each sample and divided by 10^8^ cells. The results displayed are representative of three biological experiments performed in triplicate. Values are the mean ± s.d. of three independent experiments.

### Flow cytometry

One milliliter of logarithmic cells were washed with PBS buffer and sonicated using 15 s pulses at 20% power. For each sample, 2*10^6^ cells were resuspended with ProteoStat dye (Enzo Life Sciences) diluted 1:3000 in ProteoStat assay buffer. Cells were incubated for 15 min at room temperature protected from light. Flow cytometry was preformed using Stratedigm S1000EXi and the CellCapTure software (Stratedigm, San Jose, CA). Live cells were gated (P1) by forward scatter and side scatter. Fluorescence channels for FITC (530/30) and PE-Cy5 (676/29) were used utilizing a 488 nm laser source. A total of 50,000 events were acquired for each sample. Analyses were performed using FlowJo software (TreeStar, version 10). The results displayed are representative of three biological experiments performed in triplicate.

### Confocal microscopy

One milliliter of logarithmic cells were washed with PBS buffer, sonicated using 15 s pulses at 20% power and resuspended in 50 µL of ProtesoStat dye diluted 1:250 in ProteoStat assay buffer. Cells were incubated for 15 min at room temperature protected from light. 10 µL of each sample was deposited on poly-lysine-coated glass slides (Sigma-Aldrich). Cells were imaged using Leica TCS SP8 laser confocal microscope with ×63 1.4 NA or ×100 1.4 NA oil objectives. To visualize the nuclei, Hoechst (33342; ImmunoChimestry) was used. An argon laser with 488 excitation line was used for ProteoStat (emission wavelength, 500–600 nm), and a 405 UV laser was used to excite Hoechst fluorescence (emission wavelength, 415–450 nm). The results displayed are representative of three biological experiments.

### Confocal Raman microscopy

All Raman spectroscopic and imaging experiments were performed with a laboratory-made confocal Raman microscope, details of which can be found elsewhere^41^. A He–Ne laser with an output wavelength of 632.8 nm was used for excitation. Laser power of 5 mW was used at the sample point with an exposure of 0.5 s per point. For Raman imaging experiments, a step size of 0.6 µm was used. The results displayed are representative of three biological experiments.

### Anti-adenine fibril antibodies production

Adenine at a final concentration of 8 mg/mL was dissolved in PBS at 90 °C, followed by overnight gradual cooling for the formation of adenine fibrils. These structures served as antigens in a series of seven immunization cycles in rabbit followed by purification of polyclonal IgG antibodies using protein G column. The antibodies were supplied by Adar Biotech (Rehovot, Israel).

### Indirect immunofluorescence

Eight milliliters of yeast cells at the logarithmic phase were fixed for 2 h with formaldehyde (3.7% final) and potassium phosphate buffer (100 mM final, pH 6.5), washed and resuspended in sorbitol buffer (1.2 M sorbitol and 100 mM potassium phosphate, pH 6.5). Sorbitol buffer supplemented with β-mercaptoethanol (20 mM) and zymolyase 20T (12.5 mg/mL) was added for 1.5 h at 37 °C to digest the cell wall. The cells were then fixed on poly-l-lysine-coated glass slides and permeabilized with PBT (PBS with 1% BSA and 0.05% Triton X-100). Immunofluorescence directed against adenine amyloid-like structures was performed using the anti-ADE antibody described above at a dilution of 1:200 for 2 h followed by three washes (PBT) and incubation with Cy3-conjugated anti-rabbit diluted 1:200 for an additional 1.5 h followed by three washes (PBS). DNA was stained with 4′,6-diamidino-2-phenylindole (DAPI). Cells were imaged using Leica TCS SP8 laser confocal microscope with ×63 1.4 NA oil objective. The results displayed are representative of three biological experiments.

### Reporting summary

Further information on experimental design is available in the Nature Research Reporting Summary linked to this article.

## Supplementary information

Supplementary Information

Reporting Summary

## Data Availability

Other data are available from the corresponding author upon reasonable request. A reporting summary for this article is available as a Supplementary information file.
