# Supplementary material for: Fibril formation and therapeutic targeting of amyloid-like structures in a yeast model of adenine accumulation
Source: Nat Commun. 2019 Jan 8;10:62. doi: 10.1038/s41467-018-07966-5 (PMC6325136; doi:10.1038/s41467-018-07966-5)
Supplement: Supplementary file 1 — Supplementary Information [file 41467_2018_7966_MOESM1_ESM.pdf]

Fibril Formation and Therapeutic Targeting of Amyloid-like Structures in a Yeast Model of Adenine Accumulation

D. Laor *et al*

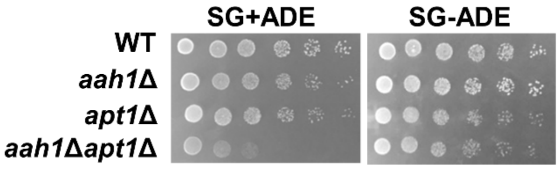

Supplementary Figure 1. Respiratory competence of the salvage mutant upon adenine feeding using glycerol as a carbon source. WT, *aah1*Δ, *apt1*Δ and *aah1*Δ*apt1*Δ strains were serially diluted and spotted on SG complete medium (2% glycerol) containing 20 mg/L adenine (SG+ADE) or on SG medium without adenine (SG-ADE).

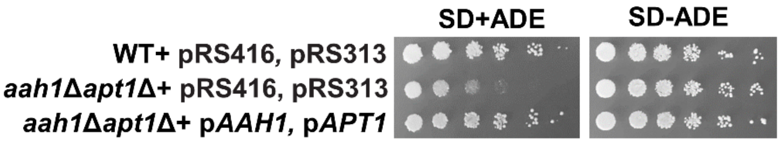

Supplementary Figure 2. ***aah1*$^{+}$ and *apt1*$^{+}$ insertion to *aah1*Δ*apt1*Δ strain restore the cell growth toxicity observed in the presence of adenine.** *aah1*Δ*apt1*Δ strain transformed with single copy plasmids carrying pRS416-*AAH1(*p*AAH1)* and pRS313-*APT1(*p*APT1)* as well as WT and *aah1*Δ*apt1*Δ strains transformed with vectors only (pRS416 and pRS313) were serially diluted and spotted on SD medium without the relevant markers (uracil and histidine respectively) containing 20 mg/L adenine (SD+ADE) or without adenine (SD-ADE).

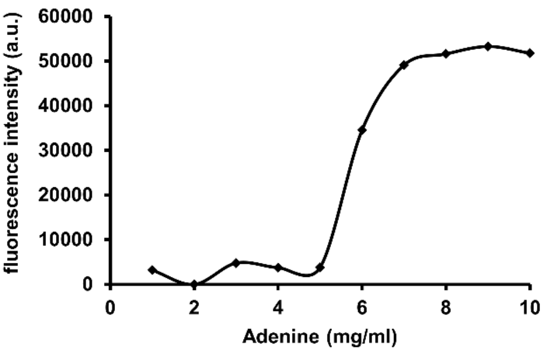

Supplementary Figure 3. Adenine self-assembly *in vitro* at different concentrations. Adenine was dissolved to a final concentration of 1 mg/ml to 10 mg/ml, as indicated. Next, 20 μM ThT (final concentration) was added following an overnight incubation and fluorescence emission endpoint measurement at 480 nm (excitation at 450 nm) was carried out.

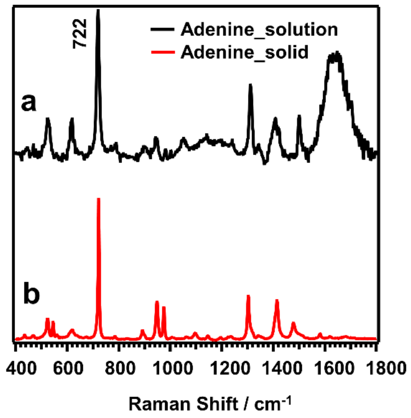

Supplementary Figure 4. Raman spectra of adenine. a, Raman band was measured using 5mg/ml of adenine in 0.5M HCl_{aq} solution. **b,** Raman band of adenine in solid from.

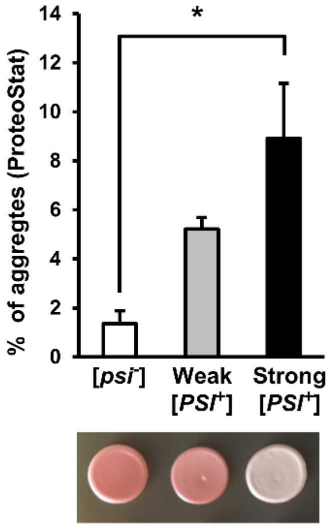

Supplementary Figure 5. *In vivo* characterization of distinct amyloid fibers formed by the yeast prion protein Sup35 by ProteoStat staining. 10µl of W303 [*psi*$^-$], weak [*PSI*$^+$] and strong [*PSI*$^+$] were spotted on YPD plate for color distinction following ProteoStat staining and analyzed by flow cytometry, showing a correlation to the level of [*PSI*$^+$]: red, pink and white respectively. **P* < 0.01 (Student's *t*-test). Values are the mean ± s.d. of three experiments.

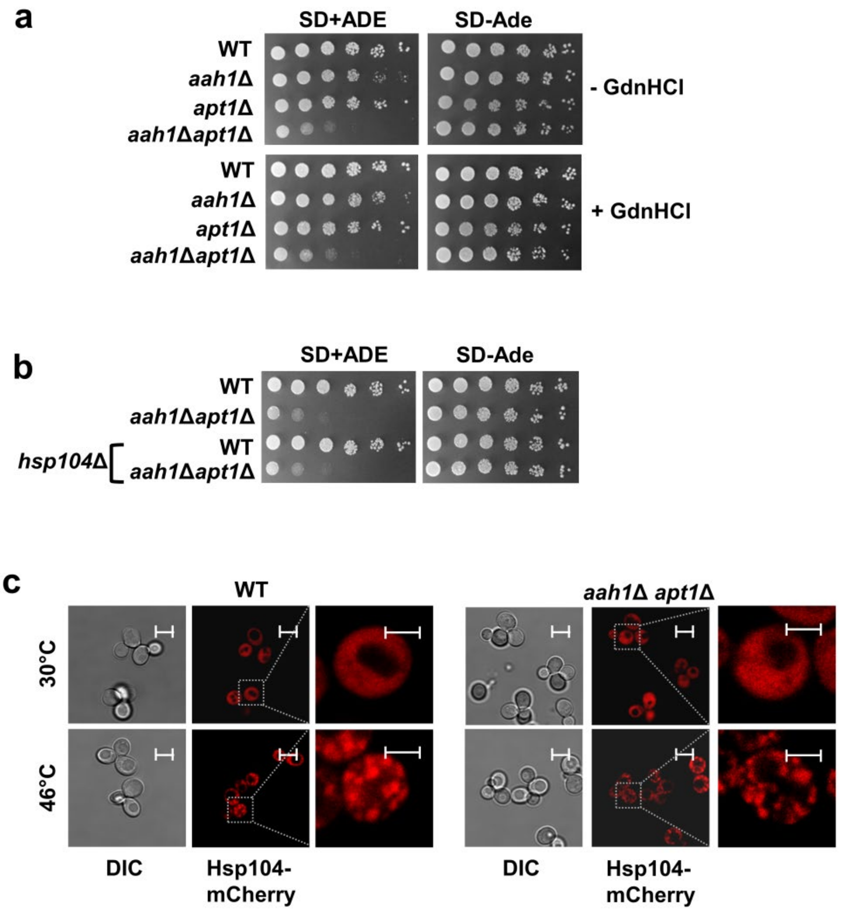

Supplementary Figure 6. The sensitivity of the salvage mutant to adenine feeding is independent of Hsp104. a, WT, *aah1*Δ, *apt1*Δ and *aah1*Δ*apt1*Δ strains were serially diluted and spotted on SD complete medium containing 20 mg/L adenine (SD+ADE) or on SD medium without adenine (SD-ADE) in the absence (-GdnHCl) or presence (+GdnHCl) of 5mM guanidine hydrochloride. **b,** WT, *aah1*Δ*apt1*Δ, *hsp104*Δ and *aah1*Δ*apt1*Δ*hsp104*Δ strains were serially diluted and spotted on SD complete medium containing 20 mg/L adenine (SD+ADE) or on SD medium without adenine (SD-ADE). **c,** Representative confocal and differential interference contrast (DIC) images of WT and Δ*aah1*Δ*apt1* cells expressing Hsp104-mCherry on SD complete medium containing 20 mg/L adenine at 30°C and following heat shock (15 minutes at 46°C). Scale bar is 5 μm. Images in the right panel of each strain/condition are magnified views of the area marked with dashed lines (scale bar is 2 μm).

a

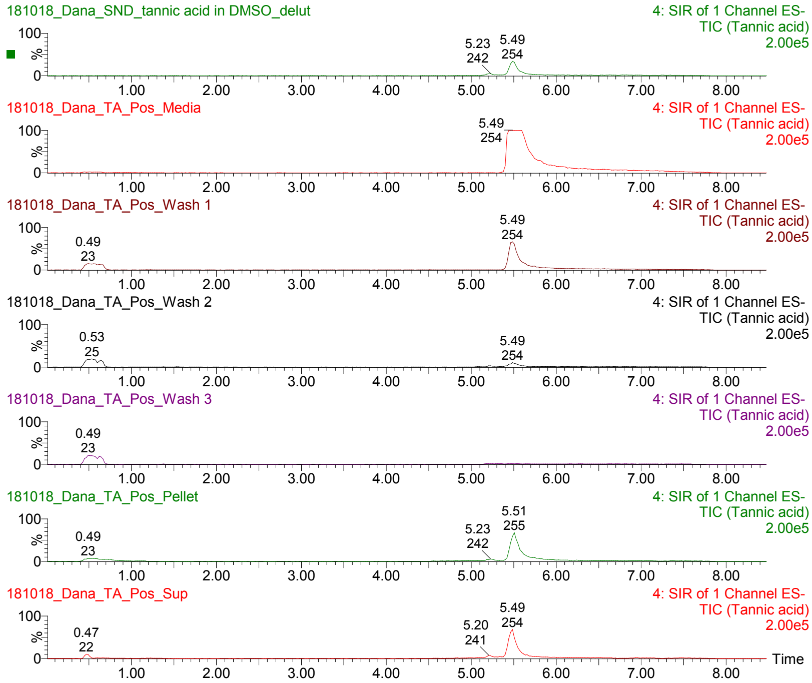
181018_Dana_SND_tannic acid in DMSO_delut
4: SIR of 1 Channel ES-
TIC (Tannic acid)
2.00e5
5.23
242
5.49
254
181018_Dana_TA_Pos_Media
5.49
254
181018_Dana_TA_Pos_Wash 1
0.49
23
5.49
254
181018_Dana_TA_Pos_Wash 2
0.53
25
5.49
254
181018_Dana_TA_Pos_Wash 3
0.49
23
181018_Dana_TA_Pos_Pellet
0.49
23
5.23
242
5.51
255
181018_Dana_TA_Pos_Sup
0.47
22
5.20
241
5.49
254
100
%
0
1.00
2.00
3.00
4.00
5.00
6.00
7.00
8.00
Time

b

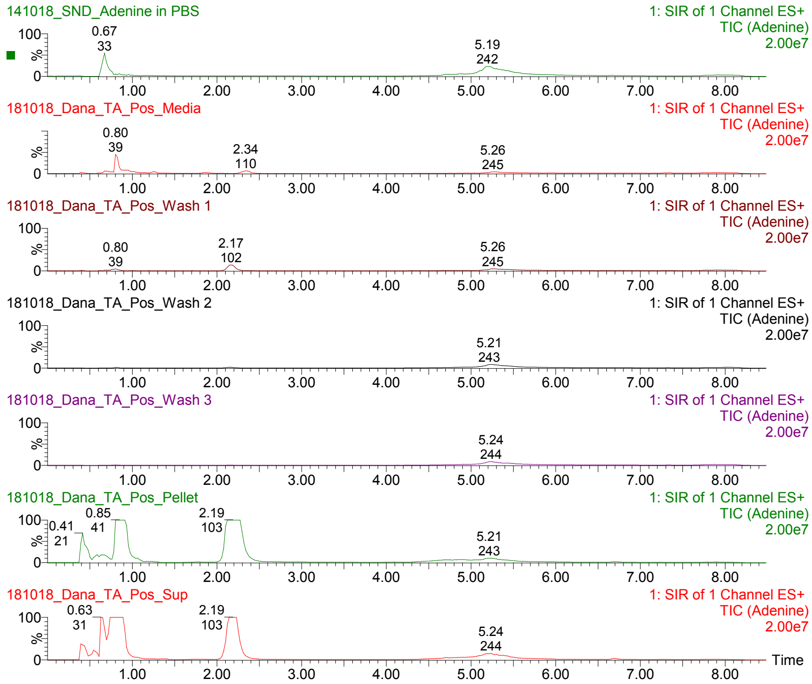
141018_SND_Adenine in PBS
1: SIR of 1 Channel ES+
TIC (Adenine)
2.00e7
0.67
33
5.19
242
181018_Dana_TA_Pos_Media
0.80
39
2.34
110
5.26
245
181018_Dana_TA_Pos_Wash 1
0.80
39
2.17
102
5.26
245
181018_Dana_TA_Pos_Wash 2
5.21
243
181018_Dana_TA_Pos_Wash 3
5.24
244
181018_Dana_TA_Pos_Pellet
0.41
21
0.85
41
2.19
103
5.21
243
181018_Dana_TA_Pos_Sup
0.63
31
2.19
103
5.24
244
100
%
0
1.00
2.00
3.00
4.00
5.00
6.00
7.00
8.00
Time

c

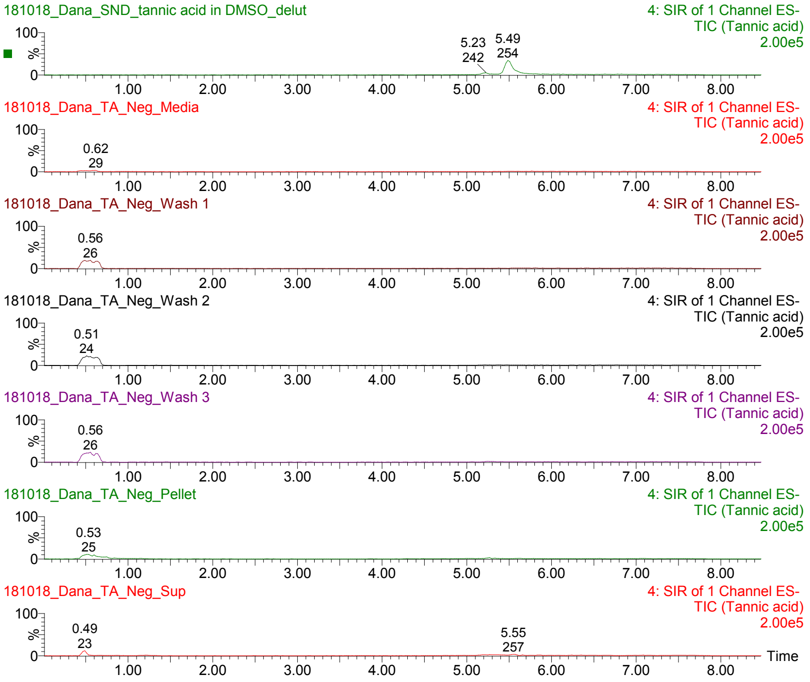

d

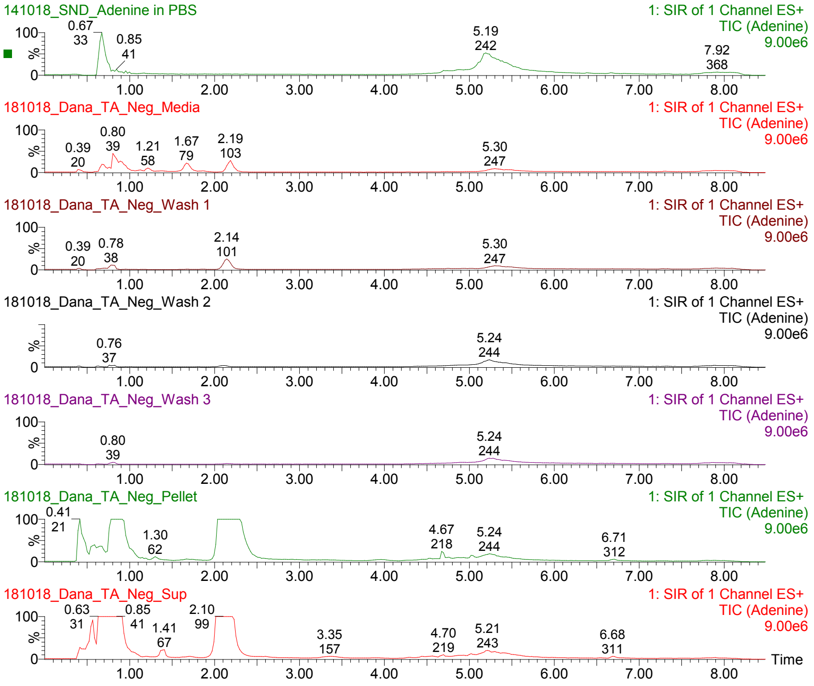

Supplementary Figure 7. TA is present inside *aah1Δapt1Δ* cells together with adenine. a-d, *aah1Δapt1Δ* cells were grown in SD medium containing adenine and TA (**a, b**) or in SD medium containing adenine without TA (**c, d**). LC-MS analysis focused on TA negatively charged molecular peak at 1700 Da, RT around 5.40 minutes (**a, c**) and on adenine positively charged molecular peak at 136.1 Da, RT around 0.70 minutes (**b, d**). Examined samples were tannic acid and adenine as standards (SND), the growth medium (Media), the washing steps (Wash1-3), the pellet (Pellet) and the supernatant (Sup).

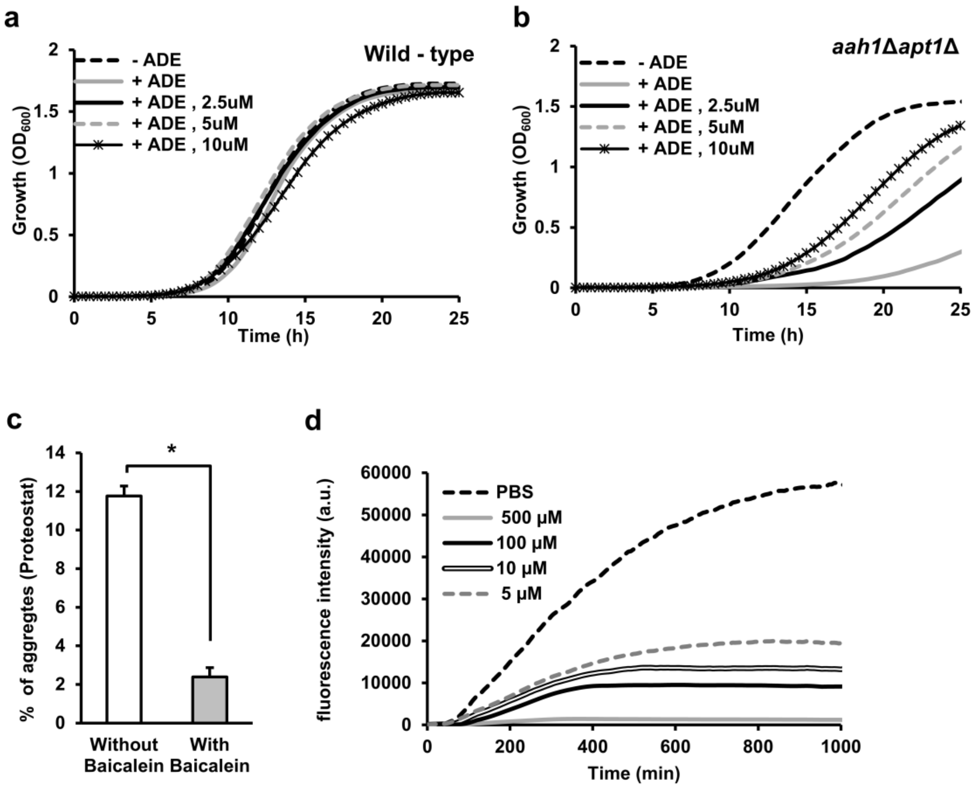

Supplementary Figure 8. Addition of baicalein rescues the toxic effect observed in the adenine salvage mutant. a-b, Growth curves of WT (**a**) and Δ*aah1*Δ*apt1* (**b**) strains in SD medium containing 2 mg/L adenine (+ADE), with or without the following concentrations of baicalein: 2.5 μM, 5 μM and 10 μM. **c,** Flow cytometry analysis of Δ*aah1*Δ*apt1* cells with and without 10 μM baicalein following ProteoStat staining. *$P < 0.01$ (Student's *t*-test). Values are the mean ± s.d. of three experiments. **d,** Adenine self-assembly *in vitro* in the presence of baicalein. ThT fluorescence emission intensity at 480 nm (excitation at 450 nm) in the presence of 8 mg/ml adenine was measured over time in the absence (PBS) and in the presence of baicalein at the following concentrations: 5 μM, 10 μM, 100 μM, 500 μM. Adenine was dissolved and treated as described in the Methods section.

Supplementary Table 1: list of strains

**Yeast Strains**	**Genetic background**	**Source**
Wild-type, BY4741	*MATa, his3-Δ1, leu2Δ-0, met15-Δ0, ura3-Δ0*	Martin kupiec's lab
apt1Δ	*MATa, his3-Δ1, leu2Δ-0, met15-Δ0, ura3-Δ0, apt1::hphMX*	Martin kupiec's lab
aah1Δ	*MATa, his3-Δ1, leu2Δ-0, met15-Δ0, ura3-Δ0, aah1:: KanMX*	Martin kupiec's lab
aah1Δapt1Δ	*MATa, his3-Δ1, leu2Δ-0, met15-Δ0, ura3*-Δ0, *aah1:: KanMX, apt1::hphMX*	This study
W303	*MATa, ade2-1, trp1-1, can1-100, leu2-3 , his3-11,15, ura3-1*	Martin kupiec's lab
74-D694, strong [*PSI*$^{+}$] [pin-]	*ade 1-14* (UGA)*, trp1-289* (UAG)*, leu2-3,112 , his3Δ -200, ura3-52*, strong [*PSI*$^{+}$] [*pin*$^{-}$] variant	Susan Lindquist 's lab
74-D694, weak [*PSI*$^{+}$] [pin-]	*ade 1-14* (UGA)*, trp1-289* (UAG)*, leu2-3,112 , his3Δ -200, ura3-52*, weak [*PSI*$^{+}$] [*pin*$^{-}$] variant	Susan Lindquist 's lab
hsp104Δ	*MATa, his3-Δ1, leu2Δ-0, met15-Δ0, ura3-Δ0, hsp104:: NAT*	This study
aah1Δapt1Δhsp104Δ	*MATa, his3-Δ1, leu2Δ-0, met15-Δ0, ura3-Δ0, hsp104:: NAT, aah1:: KanMX, apt1::hphMX*	This study
Wild-type, pRS313 pRS416	*MATa, his3-Δ1, leu2Δ-0, met15-Δ0, ura3-Δ0*, pRS313, pRS416	This study
aah1Δapt1Δ, pRS313 pRS416	*MATa, his3-Δ1, leu2Δ-0, met15-Δ0, ura3-Δ0, aah1:: KanMX, apt1::hphMX*, pRS313, pRS416	This study
aah1Δapt1Δ, pRS313 pRS416*aah1*	*MATa, his3-Δ1, leu2Δ-0, met15-Δ0, ura3-Δ0, aah1:: KanMX, apt1::hphMX*, p*APT1*, p*AAH1*	This study
Wild-type, Hsp104-mCherry	*MATa, his3-Δ1, leu2Δ-0, met15-Δ0, ura3-Δ0*, pAG415-Hsp104-mCherry	This study
aah1Δapt1Δ, Hsp104-mCherry	*MATa, his3-Δ1, leu2Δ-0, met15-Δ0, ura3-Δ0, aah1:: KanMX, apt1::hphMX*, pAG415-Hsp104-mCherry	This study

Supplementary Table 2: list of plasmids

Plasmid name	Description	Source
pUC57- *AAH1*	pUC57 derivative containing a de novo synthesis of AAH1 gene	GenScript
pUC57- *APT1*	pUC57 derivative containing a de novo synthesis of APT1 gene	GenScript
pRS313	Yeast centromere vector with a HIS3 marker	Martin kupiec's lab
p*APT1*	[pRS313] *APT1*	This study
pRS416	Yeast centromere vector with a URA3 marker	Martin kupiec's lab
p*AAH1*	[pRS416] *AAH1*	This study
pRS415	Yeast centromere vector with a LEU2 marker	Martin kupiec's lab
pAG415-Hsp104-mCherry	pAG415GPD- Hsp104-mCherry	Simon Alberti's lab

Supplementary Table 3: list of primes

Primer name	Description
Apt1_Fw	CCAACCTACTAGTAGTGGTC
Apt1_ Rev	CTACTTTGTGGCACAAAGCAG
Aah1_Fw	GACAAGCAGGCCTGTGTATTT
Aah1_ Rev	GTTCCCGAATTGTAATCTCGTG
Hsp104_Fw	CATTGAACCCTCCATCGTGGTAG
Hsp104_Rev	GAGTCATATGAGTCGCTACATTC
KanMX_Rev	CACCATGAGTGACGACTGAAT
Hyg_Rev	CATCAGCTCATCGAGAGCCTG
natMX6_ Fw	GGGGTTCACCCTCTGCGGCC
natMX6_ Rev	GGGACACTGGTGCGGTACCG
pRS306_Fw	CGCCAGCTGGCGAAGGGGGATGTGCTGCA
pRS306_Rev	ATTAGGCACCCCAGGCTTTACACTT

Supplementary Methods

ThT fluorescence endpoint measurements

Adenine was dissolved at various concentrations, ranging from 1 mg/ml to 10 mg/ml at 90°C in PBS and plated on a 96-well black plate together with 20µm ThT in PBS (final concentration). Following an overnight incubation at room temperature, ThT emission signal at 480 nm (excitation at 450 nm) was measured using a TecanTM Infinite® 200 PRO plate reader. The results displayed are representative of three biological experiments performed in triplicate.

ThT kinetic assay of adenine self-assembly inhibition

Adenine was dissolved to a final concentration of 8 mg/ml at 90°C in PBS. The solution was plated on a 96-well black plate and mixed with the inhibitor baicalein at the stated concentration, and with 20µm ThT (final concentration). Fluorescence (excitation at 450nm, emission at 480nm) was recorded over time using a TecanTM Infinite® 200 PRO plate reader. Data processing was performed using the OriginLab software. The results displayed are representative of three biological experiments performed in triplicate.

LC-MS

100 ml of cells at the logarithmic phase were centrifuged and washed three times with PBS. 1ml of the growth medium and of each wash were collected for LC-MS analysis. After the third wash, the pellet was resuspended in PBS together with acid-washed glass beads. Tubes were vortexed for 45 minutes in 4°C using disruptor-genie. Cell debris and soluble material were separated by centrifugation. Both fractions as well as the medium and washes were analyzed by MS.

The pellet was resuspended in 200 µl DMSO, heated for 5 minutes at 85°C and sonicated for 5 minutes at 40 °C. The lysis mixture was centrifuged at 6000 rpm for 10 minutes. 150 µl of the lysate were transferred to a Waters 96 well ACQUITY collection plate for LC-MS analysis. 20 µl of each sample were injected to Waters Autopurification system analytical module equipped with SQD2 MS detector at the following conditions: a. LC: Waters XSelect Peptide CSH C18 column (5µm, 4.6mm x 100mm) using a 10 minute gradient from 95:5 Water:acetonitrile (both with 0.1% formic acid) to acetonitrile (0.1% formic acid); b. MS: acquisition parameters were optimized in order to select the proper ion for selected ion monitoring (SIR) experiments. The protonated molecule of adenine $[M+H]^+$ at m/z 136.1 and deprotonated molecule of tannic acid $[M-H]^-$ at m/z 1700.1 were obtained at a cone voltage of 80 V. The results displayed are representative of three biological experiments.

Plasmids cloning

The gap-repair cloning system was used to clone by transformation the pUC57-*AAH1* and pUC57-*APT1* vectors into linearized pRS416 and pRS313 plasmids respectively that contain an appropriate homology inside the yeast cells, relying on interchanging

parts of the plasmids by homologous recombination. Candidates were confirmed by PCR using primers upstream and downstream to the insertion site.
