# Supplementary material for: Fibril formation and therapeutic targeting of amyloid-like structures in a yeast model of adenine accumulation
Source: Nat Commun. 2019 Jan 8;10:62. doi: 10.1038/s41467-018-07966-5 (PMC6325136; doi:10.1038/s41467-018-07966-5)
Supplement: Supplementary file 2 — Reporting Summary [file 41467_2018_7966_MOESM2_ESM.pdf]

nature research

Corresponding author(s): ehudg@post.tau.ac.il

Reporting Summary

Nature Research wishes to improve the reproducibility of the work that we publish. This form provides structure for consistency and transparency in reporting. For further information on Nature Research policies, see Authors & Referees and the Editorial Policy Checklist.

Statistical parameters

When statistical analyses are reported, confirm that the following items are present in the relevant location (e.g. figure legend, table legend, main text, or Methods section).

n/a	Confirmed	
☐	☒	The exact sample size (*n*) for each experimental group/condition, given as a discrete number and unit of measurement
☐	☒	An indication of whether measurements were taken from distinct samples or whether the same sample was measured repeatedly
☐	☒	The statistical test(s) used AND whether they are one- or two-sided<br>*Only common tests should be described solely by name; describe more complex techniques in the Methods section.*
☐	☒	A description of all covariates tested
☐	☒	A description of any assumptions or corrections, such as tests of normality and adjustment for multiple comparisons
☐	☒	A full description of the statistics including central tendency (e.g. means) or other basic estimates (e.g. regression coefficient) AND variation (e.g. standard deviation) or associated estimates of uncertainty (e.g. confidence intervals)
☐	☒	For null hypothesis testing, the test statistic (e.g. *F*, *t*, *r*) with confidence intervals, effect sizes, degrees of freedom and *P* value noted<br>*Give P values as exact values whenever suitable.*
☒	☐	For Bayesian analysis, information on the choice of priors and Markov chain Monte Carlo settings
☐	☒	For hierarchical and complex designs, identification of the appropriate level for tests and full reporting of outcomes
☒	☐	Estimates of effect sizes (e.g. Cohen's *d*, Pearson's *r*), indicating how they were calculated
☐	☒	Clearly defined error bars<br>*State explicitly what error bars represent (e.g. SD, SE, CI)*

Our web collection on statistics for biologists may be useful.

Software and code

Policy information about availability of computer code

Data collection	No software was used
Data analysis	OriginLab software (Origin 2016)<br>LECO ChromaTOF software (Version 4.50.8.0)<br>CellCapTure software (4.0 Version)<br>FlowJo software (TreeStar, Version 10)<br>Imaris software (version 8.4)

For manuscripts utilizing custom algorithms or software that are central to the research but not yet described in published literature, software must be made available to editors/reviewers upon request. We strongly encourage code deposition in a community repository (e.g. GitHub). See the Nature Research guidelines for submitting code & software for further information.

Data

Policy information about availability of data

All manuscripts must include a data availability statement. This statement should provide the following information, where applicable:

- Accession codes, unique identifiers, or web links for publicly available datasets
- A list of figures that have associated raw data
- A description of any restrictions on data availability

The authors declare that all of the data supporting the findings of this study are available within the article and its Supplementary Information file.

Field-specific reporting

Please select the best fit for your research. If you are not sure, read the appropriate sections before making your selection.

☒ Life sciences ☐ Behavioural & social sciences ☐ Ecological, evolutionary & environmental sciences

For a reference copy of the document with all sections, see nature.com/authors/policies/ReportingSummary-flat.pdf

Life sciences study design

All studies must disclose on these points even when the disclosure is negative.

Sample size	Sample size was determined for each experiment as was done for other experiments in the litratrature with the same protocol.
Data exclusions	No data was excluded
Replication	For each assay in the paper there was at least three biological experiments. All attempts at replication were successful.
Randomization	Since each sample from the yeast strains cells was grow in a different glass tube, no allocation is needed.
Blinding	The confocal microscopy analysis were made with the help of experts that were not aware of the meaning of the results. Other assays are not blinded since they are output of different devices and no need for blindness required or was used in other articles.

Reporting for specific materials, systems and methods

Materials & experimental systems

n/a	Involved in the study	
☒	☐	Unique biological materials
☐	☒	Antibodies
☐	☒	Eukaryotic cell lines
☒	☐	Palaeontology
☐	☒	Animals and other organisms
☒	☐	Human research participants

Methods

n/a	Involved in the study	
☒	☐	ChIP-seq
☐	☒	Flow cytometry
☒	☐	MRI-based neuroimaging

Antibodies

Antibodies used	- Anti-adenine fibril antibodies: produce for this study and supplied by Adar Biotech (Rehovot, Israel).<br>- Anti-rabbit Cy3 antibody (#111165003, Jackson Immunoresearch)
Validation	Validation using Dot Blot assay was done in our lab for the detection of adenine fibrils using the antibody mentioned above.

Eukaryotic cell lines

Policy information about cell lines

Cell line source(s)	Yeast

Authentication	All genetic manipuation were confirmed by PCR
Mycoplasma contamination	Not relavent - tYeast cells
Commonly misidentified lines (See ICLAC register)	Not relavent - Yeast cells

Animals and other organisms

Policy information about studies involving animals; ARRIVE guidelines recommended for reporting animal research

Laboratory animals	The study did not involve laboratory animals
Wild animals	The study did not involve wild animals
Field-collected samples	The study did not involve samples collected from the field

Flow Cytometry

Plots

Confirm that:

☒ The axis labels state the marker and fluorochrome used (e.g. CD4-FITC).

☒ The axis scales are clearly visible. Include numbers along axes only for bottom left plot of group (a 'group' is an analysis of identical markers).

☒ All plots are contour plots with outliers or pseudocolor plots.

☒ A numerical value for number of cells or percentage (with statistics) is provided.

Methodology

Sample preparation	1 mL of logarithmic cells were washed with PBS buffer and sonicated using 15s pulses at 20% power. For each sample, 2*106 cells were resuspended with ProteoStat dye (Enzo Life Sciences) diluted 1:3000 in ProteoStat assay buffer. Cells were incubated for 15 min at room temperature protected from light.
Instrument	Stratedigm S1000EXi
Software	CellCapTure software (4.0 Version)<br>FlowJo software (TreeStar, Version 10)
Cell population abundance	A total of 50,000 events were acquired for each sample that contained only the relevant population (confirmed by cell size and optical microscope examinations).
Gating strategy	Live cells were gated (P1) by forward scatter and side scatter. Fluorescence channels for FITC (530/30) and PE-Cy5 (676/29) were used utilizing a 488 nm laser source. Boundary between positive and negative staining is defined similarly on the Y axis (PE-Cy5) in all samples. The gating strategy for each sample is shown in the relavent figures.

☒ Tick this box to confirm that a figure exemplifying the gating strategy is provided in the Supplementary Information.
